# Patientenprofit einer präoperativen urologischen Sprechstunde

**DOI:** 10.1007/s41973-022-00176-z

**Published:** 2022-04-04

**Authors:** Ana Patricia da Silva Morais, Christina Rieger

**Affiliations:** 1 Universitätsklinik für Urologie, Freiburgstrasse 41c, 3010 Bern, Schweiz; 2 Selbstständige Urotherapeutin, Malans, Schweiz

**Keywords:** Patientengewinn, Ambulante Urologie Sprechstunde, Operatives Prozessmanagement, Pflegerische Optimierung – Qualitätsmanagement, Prozessstrukturierung Spital, Bénéfice pour le patient, Consultation ambulatoire d’urologie, Gestion des processus opérationnels, Optimisation des soins – gestion de la qualité, Structuration des processus de l’hôpital

## Abstract

**Hintergrund:**

Angesichts der Zunahme von chronischen Erkrankungen und Polymorbidität besteht ein Optimierungsbedarf in Bezug auf die Früherkennung von potenziellen Risiken und Prävention in der präoperativen Abklärung vor einer urologischen Hospitalisierung.

**Ziel:**

Durch die Interprofessionalisierung mit einer präoperativen urologischen Sprechstunde sollen potenzielle Risiken vor einer Operation erkannt und passende präventive Massnahmen eingeleitet werden.

**Fragestellung:**

Welchen Nutzen der Bedarfsabklärung können Patient*innen aus einer solchen präoperativen urologischen Sprechstunde ziehen?

**Methode:**

Es wurden eine Ist- und Soll-Analyse durchgeführt und darauf aufbauend das Konzept erstellt und umgesetzt. Zu Auswertungszwecken wurden die erhobenen Daten kontinuierlich in einer Accessdatenbank dokumentiert. Nach 9 Monaten wurden diese ausgewertet.

**Ergebnisse:**

Früherkennung und Prävention wurden nach Bedarf bei 73 Patient*innen erfolgreich umgesetzt und dadurch folgende Schritte eingeleitet: 5 Beratungsgespräche zur psychischen Unterstützung (7 %), 11 Empfehlungen für Nahrungsergänzungen (15 %), 9 Angehörigeninstruktionen (12 %), 3 Case-Management-Anmeldungen (4 %), 4 sexologische Beratungsgespräche (5 %) und 3 spezifische urotherapeutische Angebote (4 %).

**Schlussfolgerung:**

Eine prästationäre urologische Sprechstunde eignet sich, um die Risiken und Bedürfnisse der Patient*innen frühzeitig zu erkennen und präventive und beratende Massnahmen einzuleiten.

## Résumé

**Contexte:**

Face à l’augmentation des maladies chroniques et des polymorbidités, il existe un besoin d’optimiser l’identification précoce des risques potentiels et la prévention dans le cadre de la clarification préopératoire avant une hospitalisation urologique.

**Objectif:**

L’interprofessionnalisation avec une consultation urologique préopératoire doit permettre d’identifier les risques potentiels avant une opération et d’initier les mesures préventives appropriées.

**Question examinée:**

Quel bénéfice en termes de clarification des besoins les patients et patientes peuvent-ils tirer d’une telle consultation urologique préopératoire?

**Méthode:**

Une analyse de l’état actuel par rapport à l’état visé a été effectuée et utilisée comme base pour l’élaboration et réalisation du concept. Les données saisies ont été documentées en continu dans une base de données Access à des fins d’analyse. Elles ont été évaluées après neuf mois.

**Résultats:**

L’identification précoce et la prévention ont été réalisées selon le besoin chez 73 patients et patientes, ce qui a conduit à l’initiation des mesures suivantes: 5 consultations d’accompagnement psychologique (7 %), 11 recommandations de compléments alimentaires (15 %), 9 instructions aux proches (12 %), 3 inscriptions au case management (4 %), 4 consultations sexologiques (5 %) et 3 offres urothérapeutiques spécifiques (4 %).

**Conclusion:**

Une consultation urologique préstationnaire est appropriée pour une identification précoce des risques et besoins des patients et patientes, et permet ainsi l’initiation de mesures de prévention et de conseil.

## Einleitung

Angesichts der Zunahme von chronischen Erkrankungen, demografischen Veränderungen und Polymorbidität gewinnen operative Behandlung und Versorgung an Relevanz [1]. Einerseits ist es möglich, Leben zu retten und die Lebensqualität zu verbessern, andererseits ist bekannt, dass bis zu 65 % aller operativen Behandlungen zu Komplikationen führen [1, 2]. Diese Tatsachen können bei Patient*innen zu Ängsten und Verunsicherungen führen, sodass sie sich schnell unter Druck gesetzt fühlen, wenn Entscheidungen zu operativen Interventionen getroffen werden sollen. Deshalb ist es wichtig, dass sie effektiv, verständlich und vollumfänglich informiert und aufgeklärt werden [3]. Eine ärztliche Operationsaufklärung sollte die Beschreibung der Operation, die Ausführung von Alternativen, Komplikationen/Risiken, die angestrebten Ergebnisse und den prä- und postoperativen Verlauf beinhalten. Dabei sind die pflegerische und anästhesiologische präoperative Edukation sowie das Erfassen der potenziellen Risiken ebenfalls unerlässlich [4, 5]. Dies kann den Patient*innen helfen, angemessene Reaktionen und Bewältigungsstrategien auf verschiedene Situationen zu entwickeln sowie die Beteiligung am Behandlungsprozess zu verbessern [3, 6].

## Präoperative Beurteilung

Die präoperative Phase beginnt mit der Indikationsstellung einer Operation und ist beendet, wenn der/die Patient*in in den Operationssaal gebracht wird [7]. Gemäss Cheever u. Hinkle ist die präoperative Phase entscheidend betreffend Früherkennung und Prävention von möglichen Komplikationen. Zu diesem Zweck sollten die physischen, psychischen, geistigen sowie die sozialen Bedürfnisse der Patient*innen und deren Angehörigen eruiert werden [8]. Ausgehend von der Ermittlung der Bedürfnisse können der nachfolgende Behandlungsplan ausgearbeitet und so die Behandlungskontinuität garantiert werden [8]. Studien belegten, dass Patient*innen, die präoperativ gut vorbereitet werden, weniger Komplikationen intra- und postoperativ entwickeln. Zusätzlich nimmt die Aufenthaltsdauer ab, Patient*innen äussern weniger Ängste und benötigen geringere postoperative Analgesie. Aufgrund der besseren Vorbereitung werden auch weniger chirurgische Eingriffe abgesagt, und sie führt zu einer Prozessoptimierung [5, 6, 9–12].

Das „National Confidential Enquiry in Patient Outcome and Death“ (NCEPOD) veröffentlichte 2011 einen Bericht über die präoperative Beurteilung chirurgischer Patient*innen in Grossbritannien [13]. Dieser zeigte, dass die präoperative Beurteilung und die gewählten Interventionspfade eine entscheidende Rolle spielen bei der Identifizierung jener Patient*innen, die nach der Operation ein höheres Morbiditäts- und Mortalitätsrisiko aufweisen. Durch das interprofessionelle präoperative Erstgespräch mit Bedarfserhebung durch spezifische Assessments, Laboruntersuchungen und Anästhesiebesprechung ist eine frühzeitige Intervention möglich. Ebenso spielen die präoperative Physiotherapieinstruktion (Atemübung, Mobilisation nach Operation usw.) und Evaluierung des psychischen Zustandes eine grosse Rolle für die erfolgreiche postoperative Genesung [9, 13].

Studien deuten darauf hin, dass die beschriebenen Konzepte im Bereich der Anästhesie bereits hohen Stellenwert geniessen. Diese sollen auch auf weitere Gebiete der Medizin und Pflege übertragen werden [9]. Ärzteschaft, Pflege und Anästhesie haben in dieser Phase verschiedene wichtige Rollen, die sich gegenseitig ergänzen [3]. Eine Schlüsselempfehlung des „Royal College of Anaesthesists: Perioperative Medicine Vision Document“ und der „American Urological Association“ (AUA) ist der Ansatz eines multidisziplinären präoperativen Teams [4, 13].

## Praxisentwicklungsprojekt: prästationäre urologische Sprechstunde

Das Projekt wurde im Rahmen des Masterstudiums der Autorin an der Careum Hochschule für Gesundheit entwickelt und an der urologischen Universitätsklinik des Inselspitals Bern im November 2020 implementiert. Die Klinik verfügt über 45 Betten und behandelt pro Jahr rund 2000 Patient*innen stationär und 10.000 ambulant [14]. Prävention und die ganzheitliche Sicht auf Patient*innen soll an Priorität gewinnen [15]. In diesem Sinne und basierend auf dem biopsychosozialen Modell nach Engel wurde das Praxisentwicklungsprojekt der präoperativen urologischen Sprechstunde entwickelt. Dabei steht nicht das Krankheitsbild im Zentrum, sondern der/die Patient*in [1, 16, 17].

Die prästationäre urologische Sprechstunde startete mit Patient*innen, welche eine transurethrale Resektion der Blase (TUR-B) und/oder Prostata (TUR-P) erhielten. Die Absicht war, eine Patientengruppe zu wählen, welche nicht einen offenen chirurgischen Eingriff hat, um die Komplexität bei Projektstart niedrig zu halten, um die Prozesse zu entwickeln. Die Vorteile der präoperativen urologischen Sprechstunde wurden rasch erkannt, und diese wurde auf folgende Operationen erweitert: Zystektomie mit Ileumconduit, Pouchanlage oder Ersatzblase, retroperitoneale Lymphadenektomie, Pyeloplastik, Harnleiterneuimplantation oder Nephrektomie. Zukünftig sollen auch Patienten mit einer radikalen Prostatektomie in das Projekt integriert werden, da diese bis dato das Angebot einer separaten spezialisierten Sprechstunde durch eine „nurse practioner“ wahrnehmen konnten.

## Ziele und Fragestellung

Durch die präoperative urologische Sprechstunde sollen potenzielle Risiken vor einer Operation erkannt und präventive Massnahmen eingeleitet werden. Folgende Fragestellung wurde formuliert: Welchen Nutzen der Bedarfsabklärung können Patient*innen aus einer solchen präoperativen urologischen Sprechstunde ziehen?

## Methode

Die Durchführung einer Ist- und Soll-Analyse bildete die Grundlage für die Konzeptionierung des Projektes. Pro Quartal fanden Sitzungen mit dem Projektteam statt, um den Standort des Projektes zu diskutieren und eventuelle Anpassungen durchzuführen. Zusätzlich wurden Anpassungen anhand von Feedbacks durch die Mitarbeitenden und Patient*innen aufgenommen und integriert.

Zur fortlaufenden Evaluation des Projektes wurden während der 9 Monate von November 2020 bis Juli 2021 die Daten der präoperativen urologischen Sprechstunde und die Antworten der Patient*innen auf die Frage: *Wie zufrieden sind Sie mit dem Inhalt und Durchführung der Sprechstunde gewesen?* prospektiv in einer Accessdatenbank erfasst. Folgende Indikatoren wurden ermittelt: demografische Daten, Operationseingriffe, Patientenzufriedenheit, präoperative Angebote sowie Screenings/Assessments.

## Inhalte der präoperativen urologischen Sprechstunde

In Frage kommende Patient*innen werden maximal 3 Wochen vor der Operation für die präoperative urologische Sprechstunde aufgeboten. Dabei müssen folgende Voraussetzungen gegeben sein:

- Patient*in ist mit der präoperativen urologischen Sprechstunde einverstanden;
- Operationsaufklärung und Voruntersuchungen/Staging wurden durchgeführt.

In der präoperativen urologischen Sprechstunde findet ein umfassendes Pflegegespräch statt. Dieses Gespräch beinhaltet die Erfassung der eigenen Medikamente und des präoperativen Medikamentenmanagements, die Erklärung möglicher Untersuchungen und/oder weiterer diagnostischer Massnahmen sowie eine Erläuterung des Ablaufs vom Eintritts- bis zum Austrittstag. Zugleich werden standardmässig verschiedene Screenings/Assessments genutzt, um die Bedürfnisse der Patient*innen abzuklären. Studien zeigten, dass Krebspatienten häufig an Stress und Mangelernährung leiden [18]. Aus diesem Grund empfiehlt das „National Comprehensive Cancer Network“ (NCCN), dass alle Krebspatienten routinemässig auf Stress untersucht werden. Dafür wird das Disstressthermometer benutzt. Bei dem Instrument handelt es sich um ein Selbstauskunftsinstrument, das eine Bewertungsskala von 0–10 verwendet [18]. Für das Ernährungsscreening wird wiederrum das „nutritional risk screening“ (NRS) empfohlen [19]. Anhand des Ergebnisses und des Bedarfs der einzelnen Patient*innen können eine präoperative Anmeldung für psychische Unterstützung durchgeführt, Ernährungsberatung involviert oder nur Nahrungsergänzung empfohlen werden.

Durch die Pflegeanamnese erfolgen zusätzlich auch die Erhebung zum Dekubitus- und Sturzrisiko sowie die Erfassung des sozialen Status und anderer individueller Bedürfnisse. So können schon präventive Massnahmen vor dem Eintritt vorgenommen werden.

Darüber hinaus findet eine Patienten- und Angehörigenedukation statt bezüglich Instruktion von Physiotherapieübungen, wie z. B. Atemübungen, Mobilisation nach der Operation und Übungen zur Aktivierung des Kreislaufs. Die Physiotherapieübungen werden bei allen Operationen ausser bei kleinen Eingriffen instruiert. Grund dafür ist, dass der Spitalaufenthalt, die Operationsdauer und das Risiko venöser Thromboembolien bei den kleinen Eingriffen geringer ist [20].

Im Rahmen der präoperativen urologischen Sprechstunde findet auch parallel das Anästhesieaufklärungsgespräch statt. Bei offenen Fragen oder falls eine Operationsaufklärung noch offen ist, wird zusätzlich der/die Urolog*in involviert.

## Ergebnisse

### Demografische Daten und Operationen

Von den 73 Patient*innen (*n* = 73) sind 58 Männer und 15 Frauen (Tab. 1).

**Table 1:** 

Merkmale	Wert
Alter (Jahre), Mittelwert (sd)	66,0 (13)
*Geschlecht, n (%)*	
Frau	15 (21)
Mann	58 (80)
*Operation, n (%)*	
TUR‑P	31 (43)
TUR‑B	28 (39)
Pyeloplastik	1 (1)
Ileumconduit	2 (3)
Pouchanlage	1 (1)
Nephrektomie	7 (10)
Retroperitoneale Lymphadenektomie	2 (3)
Harnleiterneuimplantation	1 (1)

### Patientenzufriedenheit und präoperative Angebote

Die Auswertung der Daten zeigte eine hohe Patientenzufriedenheit mit dem Inhalt und der Durchführung der präoperativen urologischen Sprechstunde (Tab. 2). Des Weiteren ist ersichtlich, dass einige präoperative Angebote wahrgenommen wurden (Tab. 3). Vorherige Daten dazu existieren nicht, daher ist ein Vergleich nicht möglich.

**Table 2:** 

Merkmale	*n* (%)
*Patientenzufriedenheit*	
Mehrheitlich zufrieden	44 (60)
Sehr zufrieden	26 (36)
Mehrheitlich unzufrieden	3 (4)

**Table 3:** 

Merkmale	*n* (%)
*Case-Management*	
Angemeldet	3 (4)
Kein Bedarf	70 (96)
*Sozialdienst*	
Abgelehnt	3 (4)
Kein Bedarf	70 (96)
*Urotherapeutische Angebote*	
Kein Bedarf	68 (94)
IC	2 (3)
Pouchanlage Beratung	1 (1)
Sexologie	4 (6)
Angehörigeninstruktion	9 (12)
Patientenedukation	14 (19)
Nahrungsergänzung empfehlen	11 (15)
*Anmeldung für psychische Unterstützung*	
Kein Bedarf	45 (61)
Abgelehnt	23 (32)
Seelsorge	3 (4)
Psychoonkologie	1 (1)
Psychologie	1 (1)
*„Nutrition risk score“*	
0	29 (40)
1	33 (45)
2	11 (15)
*Disstressthermometer*	
0–3	45 (61)
4–6	25 (34)
7–9	3 (4)

Die Analyse der Daten zeigte, dass die präoperativen Angebote eher bei grösseren Operationen wahrgenommen werden. So wurde z. B. bei Zystektomiepatient*innen (Ileumconduit und Pouchanlage) eine spezifische urotherapeutische Beratung hinzugezogen. Da diese Eingriffe erst seit Mai 2021 in die prästationäre urologische Sprechstunde integriert sind und daher wenige Daten zur Verfügung stehen, sind diese nur bedingt aufschlussreich. Dessen ungeachtet konnten anhand von den Screenings/Assessments Massnahmen präoperativ eingeleitet werden. Beim Disstressthermometer gaben 28/73 (38 %) der Patient*innen einen Disstress zwischen 4 und 9 an (s. Tab. 3). Von diesen 28 nahmen 5 Patient*innen das Angebot für psychische Unterstützung wahr, die restlichen 23/73 (32 %) lehnten diese ab. Von den 5 Patient*innen hatten 3 eine TUR‑B, einer eine TUR‑P und ein anderer eine Nephrektomie.

Insgesamt fand bei 14/73 (19 %) Patient*innen die Patientenedukation statt. Bei 9 davon wurde die Patientenedukation im Beisein der Angehörigen durchgeführt. Die Edukation war im Rahmen der Restriktionen aufgrund des SARS-CoV-2-Virus teilweise limitiert, da die meisten Patient*innen keine Angehörigen in die prästationäre urologische Sprechstunde mitbringen durften.

Das Case-Management wird auf der urologischen Klinik meist bei komplexen medizinischen und psychosozialen Patientensituationen eingeschaltet, welche Spitex, Rehabilitation oder andere Anschlusslösungen nach dem Spitalaustritt erfordern. Mit der präoperativen urologischen Sprechstunde war es möglich, bei 3 Patient*innen diese Dienste schon vorgängig anzumelden. Bei allen 3 Fällen ging es darum, Unterstützung für zu Hause zu organisieren, z. B. Spitex und/oder Mahlzeitendienst nach dem Spitalaustritt.

Das Angebot für sexologische Beratung wurde von 4 Patient*innen gewünscht, 1‑mal im Rahmen einer TUR‑P, 2‑mal bei Nephrektomie und 1‑mal bei der retroperitonealen Lymphadenektomie.

## Diskussion

### Patientenzufriedenheit und emotionale Belastung

In Bezug auf das Outcome, gesundheitsökonomische Gründe sowie der Patientenzufriedenheit des Spitalaufenthaltes ist ein effektives Austrittsmanagement elementar. In der heutigen Zeit werden die meisten Patient*innen bereits frühzeitig entlassen, mit dem Kernpunkt, sich Zuhause vollständig von der Operation zu erholen. Wichtige Informationen und notwendige Instruktionen müssen in den individuell zeitlich angepassten Pflegeprozess aufgenommen und patientenorientiert abgeben werden [21] – ein Grund mehr, diese Beziehung vorgängig aufzubauen. Da diese Beziehung in der präoperativen urologischen Sprechstunde aufgebaut wird und eine umfassende Informationsgabe stattfindet, ist dies ein möglicher Grund für die hohe Patientenzufriedenheit mit der prästationären urologischen Sprechstunde.

Zusätzlich ist bekannt, dass die Patient*innen viele Unsicherheiten und Ängste vor einer Operation haben, was wiederum zu Komplikationen und negativen emotionalen Reaktionen führen kann [3, 22]. Die Evaluation von der präoperativen urologischen Sprechstunde bestätigt dies. Einen Disstresslevel zwischen 1 und 3 gaben 42/73 an und über 4 gaben 28/73 (38 %) an. Von den 28 nahmen 5 ein Angebot für psychische Unterstützung an, und 23 lehnten es ab. Es soll angemerkt werden, dass unter den 45/73 (61 %) sich mehrheitlich Patient*innen mit TUR‑B und TUR‑P befanden. Im Falle einer erhöhten Integration von Patient*innen mit grösseren/komplexeren Operationen in die präoperative urologische Sprechstunde könnte auch ein Anstieg der angenommenen psychologischen Unterstützungsangebote vorkommen.

### Prävention, Früherkennung und Patientenedukation

Die vorliegende Arbeit wurde geleitet durch die Frage, ob in der präoperativen urologischen Sprechstunde Risiken erkannt werden können, um präoperative Massnahmen frühzeitig einzuleiten. Dies ist möglich, indem der physische, psychische und emotionale Zustand anhand von Screenings, Assessments und Gesprächen bewertet wird [5, 9]. In der präoperativen urologischen Sprechstunde konnten mehrere präoperative Angebote und Massnahmen initiiert werden; z. B. die Urotherapeutin für die Stomamarkierung und Stomaaufklärung bei der Zystektomie und der Anlage eines Ileumconduits, aber auch die Schulung zum sauberen intermittierenden Katheterismus bei der Pouchanlage. Auch sexologische Beratungen konnten vorgängig initiiert werden, weil der Bedarf in der Pflegeanamnese erfragt worden war. Mit der Hilfe des „nutrition risk score“ konnte bei allen Patient*innen, die einen Wert von 2 aufwiesen, prophylaktisch eine Nahrungsergänzung empfohlen und gestartet werden. Es muss jedoch berücksichtigt werden, dass insgesamt 59/73 (81 %) der Patient*innen kleinere Operationen hatten.

Die Patientenedukation erfolgte, wie vorgängig erklärt, standardmässig. Wie Studien belegten, ist die genannte Patientenedukation für eine Minimierung der postoperativen Komplikationen und Stress sowie auch eine Verbesserung der postoperativen Genesung relevant [4, 23]. Die Projektphase zeigte, dass in der präoperativen urologischen Sprechstunde weiteres Entwicklungspotenzial bezüglich Informationsabgabe/Patientenedukation existiert. Ein Ziel ist, in Zukunft verschiedene Informationsmethoden zu entwickeln, um die Patientenedukation im präoperativen Setting zu optimieren; z. B. die Nutzung von digitalen Medien, Broschüren oder Peeraustausch mit anderen Betroffenen.

## Schlussfolgerung

Die Erfahrungen und Ergebnisse von diesem Projekt zeigen, dass die präoperative urologische Sprechstunde der richtige Ansatz ist, um Patient*innen vor einem urologischen Eingriff umfassend zu informieren, potenzielle Risiken zu erkennen und präventiv zielführende Massnahmen einzuleiten. Die Auswertung zeigt, dass es möglich ist, im Vorfeld Patientenaufklärung, Angehörigeninstruktion und notwendige Angebote wie Ernährungsberatung, Kontinenz- und Sexualberatung oder psychologische Unterstützung zu initiieren. Es ist ausserdem ersichtlich, dass die Zufriedenheit der Patient*innen mit der präoperativen urologischen Sprechstunde hoch ist. Daher sollten das Angebot ausgeweitet und eine grössere Anzahl von Patient*innen einbezogen werden, denen eine komplexe Operation bevorsteht. Diese werden von der präoperativen urologischen Sprechstunde am meisten profitieren.

Nach dieser Pilotphase sind sicher weitere Untersuchungen zur Beurteilung der Prozesse und der Wertigkeit der präoperativen Sprechstunde notwendig.
